# Supplementary material for: Phytophagy impacts the quality and quantity of plant carbon resources acquired by mutualistic arbuscular mycorrhizal fungi
Source: Nat Commun. 2024 Jan 27;15:801. doi: 10.1038/s41467-024-45026-3 (PMC10821877; doi:10.1038/s41467-024-45026-3)
Supplement: Supplementary file 1 — Supplementary Information [file 41467_2024_45026_MOESM1_ESM.pdf]

Supplementary information.

Table S1. The treatments in each side of the split root system. Each system consisted of two pots, with half of the roots in each and either Arbuscular mycorrhizal fungi (AMF) or plant-parasitic nematodes (PPN; specifically the potato cyst nematode, *Globodera pallida*).

Treatment	Pot 1	Pot 2
Control	Control	Control
PPN / Control	PPN	Control
AMF / Control	AMF	Control
PPN + AMF	AMF+PPN	AMF+PPN
PPN / AMF	AMF	PPN

Table S2: Sequencing alignment statistics to host, AM fungi and PPN genomes. RNA was extracted from root tissue inoculated with either AM fungi, plant-parasitic nematodes (PPN; specifically the potato cyst nematode, *Globodera pallida),* or none, and sequenced. Treatment labels of "/" indicate the different inocula on either side of the split root, whilst "+" indicates inocula together on both sides of the root. RNA sequencing reads were aligned to reference genomes, with the following percentage alignments.

			Percentage of reads aligned to reference genomes		
Treatment	Root sample	Total reads	*S. tuberosum*	AM fungi	Potato cyst nematode
AMF / Control	Control 1	32,140,859	86.7	-	-
AMF / Control	Control 2	49,359,369	85	-	-
AMF / Control	Control 3	39,917,995	86.2	-	-
AMF / Control	Control 4	32,126,140	86.4	-	-
AMF / Control	AM fungi 1	35,083,366	74	6.2	-
AMF / Control	AM fungi 2	36,600,615	73.9	6.2	-
AMF / Control	AM fungi 3	33,208,275	75.7	7.5	-
AMF / Control	AM fungi 4	49,794,528	83.7	5.3	-
PPN + AMF	PPN+AMF1	80,616,782	79.6	4.9	5.3
PPN + AMF	PPN+AMF2	32,356,841	79.1	7.4	8.6
PPN + AMF	PPN+AMF3	37,852,470	78.5	7.8	5.8
PPN + AMF	PPN+AMF4	31,018,508	79.6	7.7	6.1
PPN / AMF	AMF1	31,437,759	86.1	7.6	-
PPN / AMF	AMF2	30,125,698	84.6	7.5	-
PPN / AMF	AMF3	30,315,337	86.2	7.5	-
PPN / AMF	AMF4	39,142,235	86.5	7.4	-

Table S3: qPCR primers for the quantification of AM fungal genes. Genes related to AM fungal transport of carbon or nutrients were obtained from the transcriptome data via filtering based on putative annotations from BLAST and gene ontology searches. Due to relatively low read counts for AM genes that may potentially reduce the reliability of RNA sequencing derived results, qPCR was performed on RNA collected from mycorrhizal colonised roots to confirm transcriptomic data.

Gene target	Forward primer	Reverse primer	Tm	Product size (bp)
SUC1	CGGGATGAGGCCAACAAGAT	TGCAACATTCTTCCTGCCCA	60	164
MST2	GGCTTTCCAACAGTTGACCG	CGATCTATCCAAACTATTGCAGGA	60	163
MST3	ATCGTTGGGGACGTAGACCA	AACAGCTAAGGAGCTTGCTTTATT	60	164
MST4	TCTTCACGCGTCGCTGATAC	CGGAACGACTACACTGGTCA	60	168
PT1	TTGAAAGTGTCGGCCTCCAT	CCGCATAATGCTGAAGCCAC	59	140
PT2	AGCAAGCTGGTCGGATTTCA	TGTGTGTCAGCGTAGCCAAT	59	159
PT3	ATGATGGCGGCCGTTTTTG	AGTGCGATTGTAGCGGGAAT	60	170
PT4	CGTTTAACAATTCCTGAAACTCCA	ACTTTTCCATTTTCCCATTGTCCA	59	153
Cu transporter	AGATCGATTGCAGTGAAAACTCT	CTACGATCAATTGTCCTGTAGTCA	59	140
Sulfate transporter	CAGCGGTTGCAATATCGGC	GCCCGCTCCCAATTGAATAC	58	161
Zn/Fe permease 1	TCGACAACGAACATCCGCTA	TGCAGCCATCGCTAATCCAT	59	148
Zn/Fe permease 2	GCACTTCACAATTTTCCTGAGGG	CCAACGTGATCCGGTTGAAT	60	156
Zn/Fe permease 3	GCATTTGCTTCAGGACTGGC	ACATTACTCCAGCACCAAGTGA	60	151

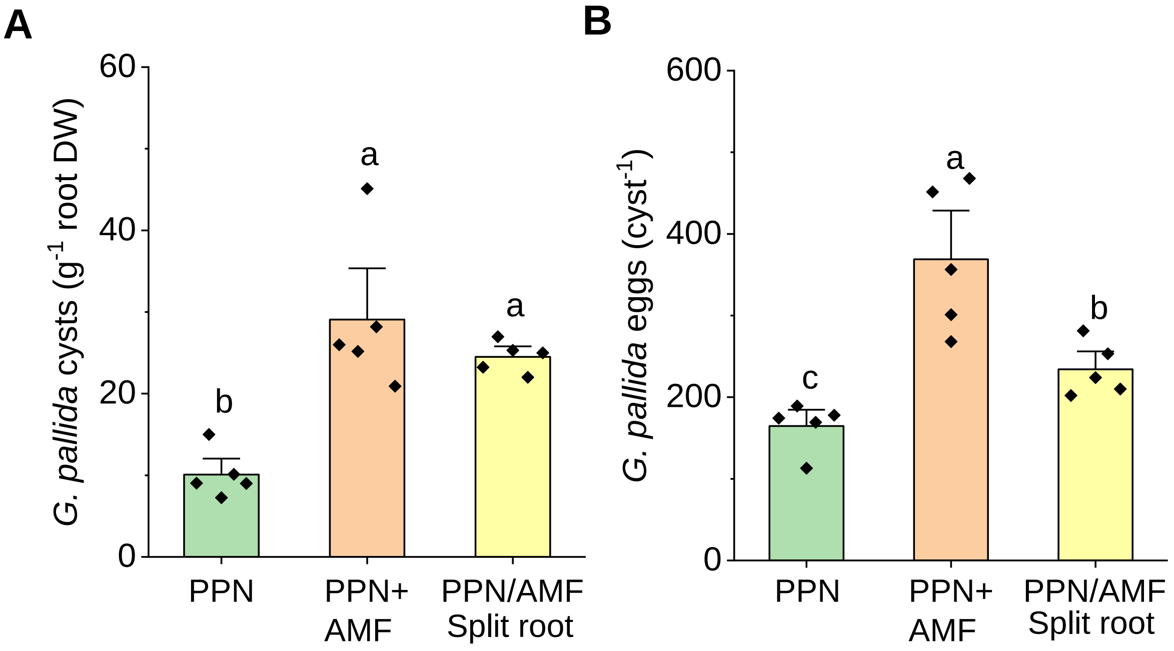

Fig S1: The impact of AM fungi on plant-parasitic nematode infection of *S. tuberosum* roots. Data indicate (a) the number of plant parasitic nematode (PPN; *Globodera pallida*) cysts/g soil and (b) the number of PPN eggs/cyst from hosts that either had only *G. pallida* and no AMF (PPN), AM fungus inoculated on the same root tissues as *G. pallida* (PPN+AMF), or AM fungus inoculated on distal root tissues to *G. pallida* (PPN/AMF). Bars represent five biological replicates with standard error of the mean. Different letters denote significance (one-way ANOVA, Tukey's Honest Significant Difference test, $p < 0.05$).

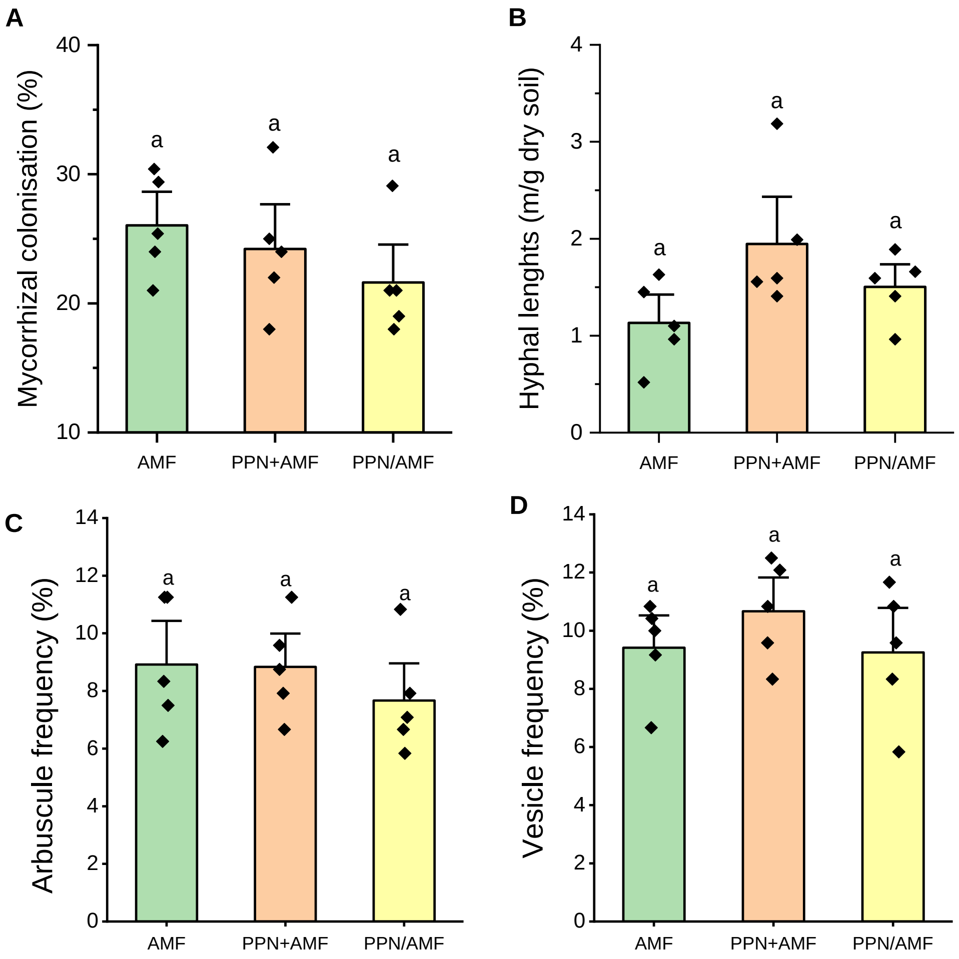

Fig S2: The impact of plant-parasitic nematode infection of *S. tuberosum* roots on AM fungal colonisation and hyphal extension. Data indicate (a) the percentage AM fungal colonisation of *S. tuberosum* roots, (b) the hyphal lengths in the soil by AM fungus, (c) arbuscule frequency within roots, and (d) vesicle frequency within roots, in the absence and presence of the plant-parasitic nematode *G. pallida* (PPN). Treatments investigate the impact of inoculating the symbionts locally or distally on the same root systems, which are indicated by x-axis labels (control = no symbiont, '/' = roots split between the two stated root compartments, '+' = both symbionts together in the same root compartments). Bars represent five biological replicates with standard error of the mean. Different letters denote significance (one-way ANOVA, Tukey's Honest Significant Difference test, $p < 0.05$).

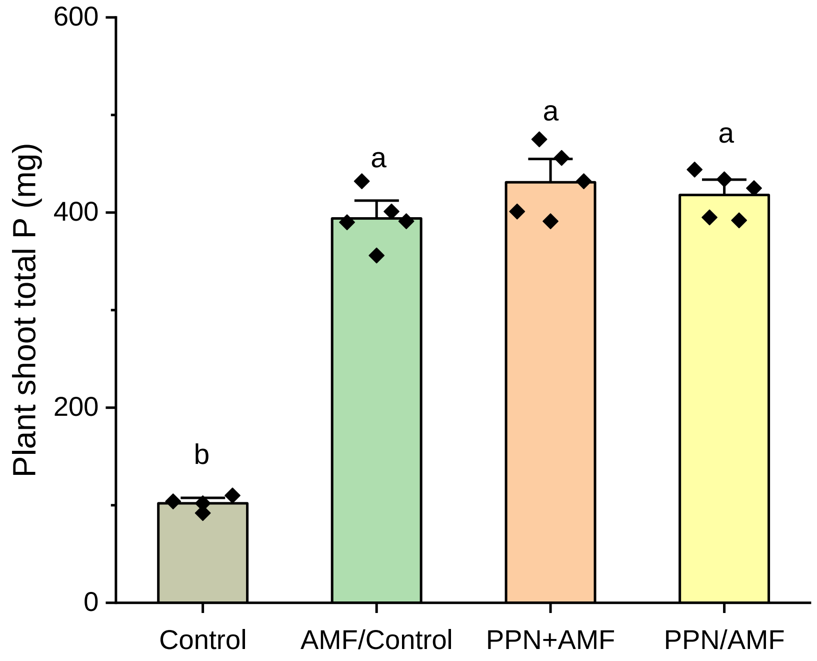

Figure S3: Plant shoot P. Total shoot P (mg) was quantified to determine the potential ramifications of the double dosage of AM fungal inocula in the PPN+AMF treatment from inoculation in both sides of the split-root setup. Treatments investigate the impact of inoculating AM fungi and plant-parasitic nematodes (PPN; *G. pallida*) symbionts locally or distally on the same root systems, which are indicated by x-axis labels (control = no symbiont, '/' = roots split between the two stated root compartments, '+' = both symbionts together in the same root compartments). As a result of this, PPN/AMF received one dosage of AM fungal inocula whereas PPN+AMF received a double dosage. Bars represent five biological replicates with standard error of the mean. Different letters denote significance (one-way ANOVA, Tukey's Honest Significant Difference test, $p < 0.05$).

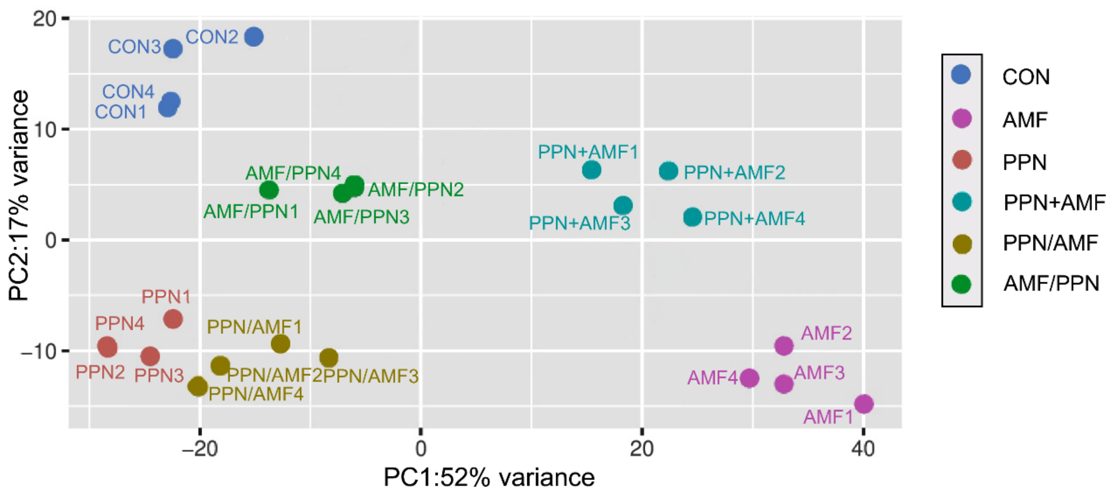

Fig S4. PCA plot of *S. tuberosum* root gene expression when inoculated with AM fungi and plant-parasitic nematodes. Samples groups are asymbiotic control (CON), AM fungus only (AMF), AM fungus and PPN infected tissue (PPN+AMF), AM fungal-colonised roots that were separate and distal from PPN-infecting the same host (AMF/PPN) and PPN-infected roots that were separate and distal from AMF-colonised roots from the same host (PPN/AMF). “Con” and “AMF” tissues were from the same host but separated by the split-root design.

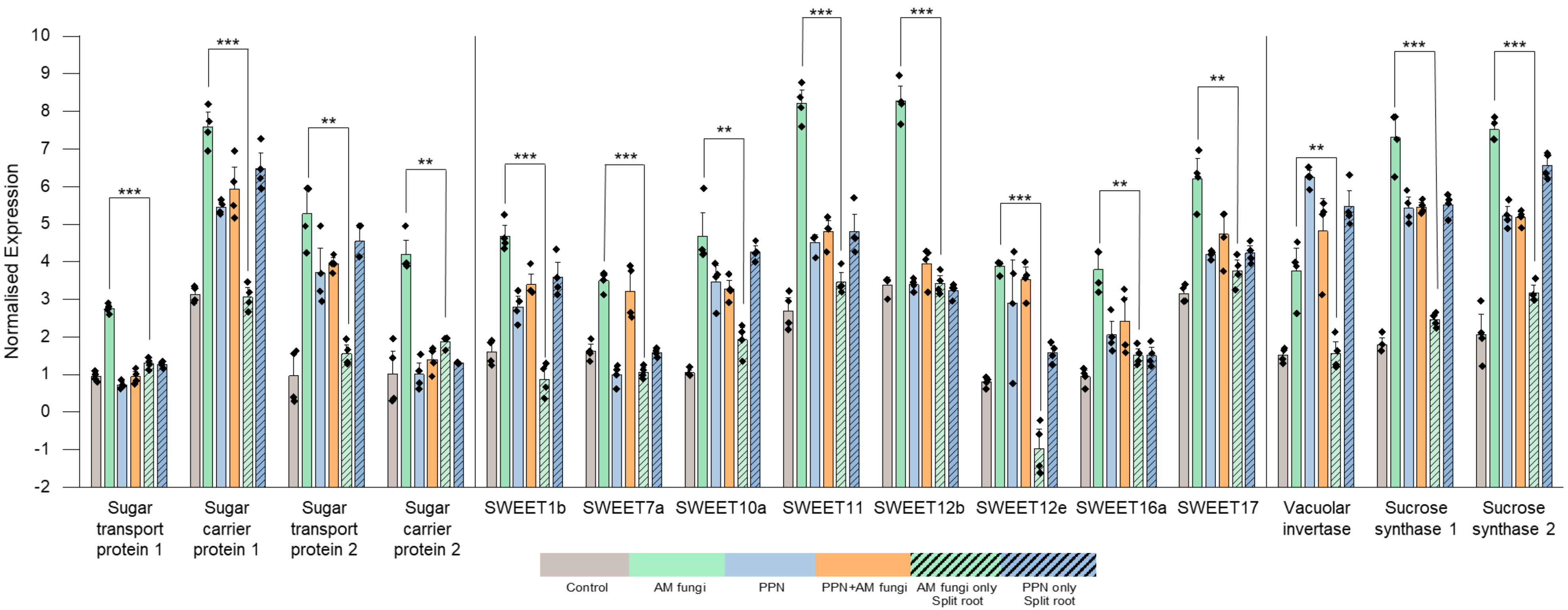

Fig S5: Differential expression of *S. tuberosum* genes related to hexose/sucrose transport in AM-colonised roots with distal PPN infection. Normalised expression (logCPM) of host genes when AM fungi and plant parasitic nematodes (PPN; *G. pallida*) are colonising/infecting root tissues. Treatments explore the potential implications of single (i.e. AM fungi, PPN) and co-colonisation of both symbionts either in the same root compartment (PPN+AM fungi) or distally separated symbionts in a split-root system (e.g. AM fungi only split root). Bars represent four biological replicates with standard error of the mean. Statistical analysis through DESeq2. Asterisk indicate the significance of upregulated genes in AM-colonised roots in the absence of PPN infection of the root system. ** = p<0.01, *** = p<0.001.

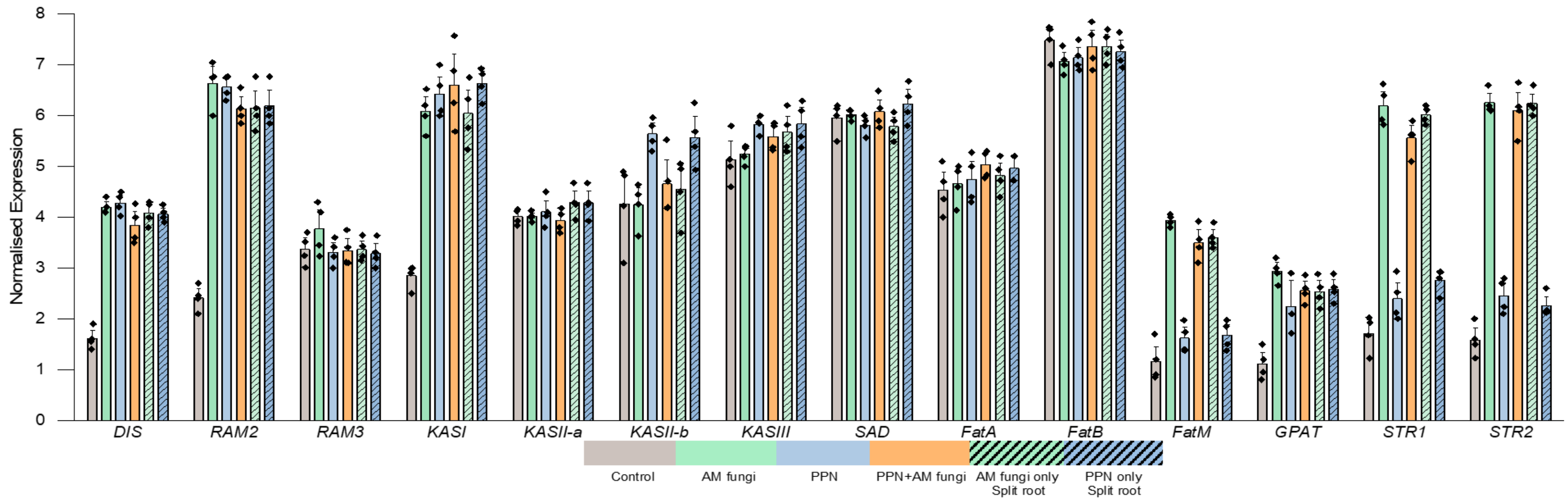

Fig S6: The expression of *S. tuberosum* genes related to fatty acid biosynthesis/transport in AM-colonised roots with distal PPN infection. Normalised expression (logCPM) of host genes when AM fungi and plant parasitic nematodes (PPN; *G. pallida*) are colonising/infecting root tissues. Treatments explore the potential implications of single (i.e. AM fungi, PPN) and co-colonisation of both symbionts either in the same root compartment (PPN+AM fungi) or distally separated symbionts in a split-root system (e.g. AM fungi only split root). Bars represent four biological replicates with standard error of the mean.

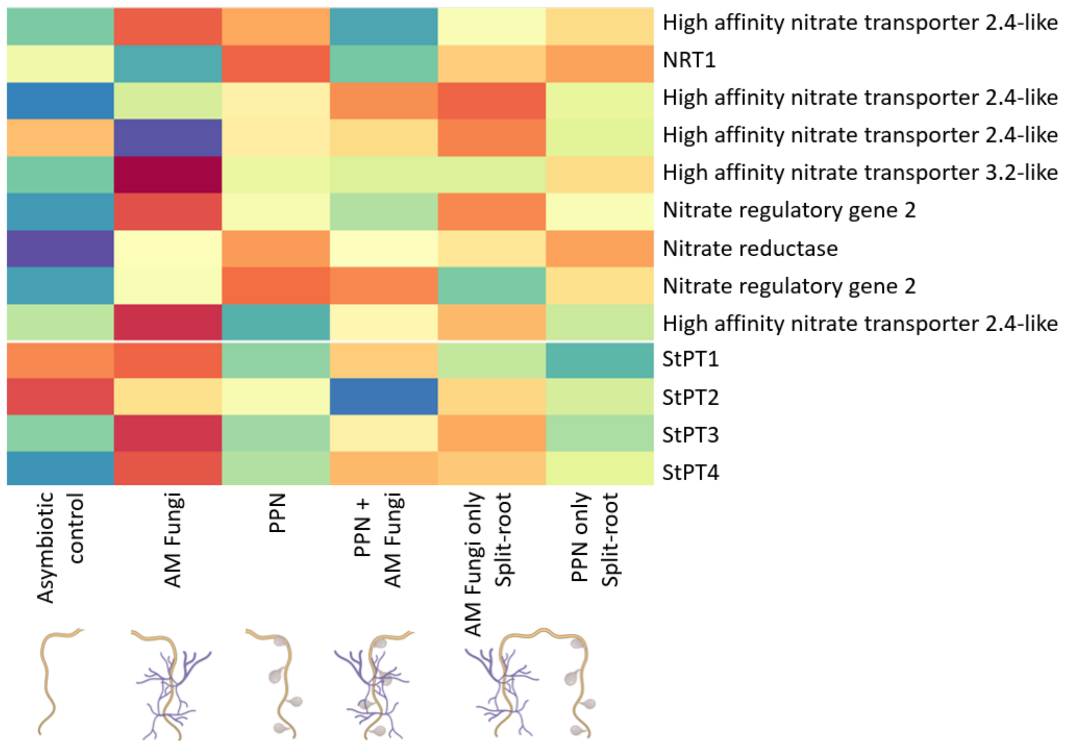

Fig S7. The expression of genes related to the movement of nitrate and phosphate in host roots. RNA from host roots were sequenced and differential genes were filtered for genes related to the transport of nitrogen and phosphate, due to their potential involvement in the intake and movement of AMF-acquired nutrients. Data is presented as expression counts of each gene (row) on a relative scale (red=higher expression and blue=lower expression). Treatments are asymbiotic control roots and AM fungal-only colonised roots from the same plant, joint AM fungi and plant-parasitic nematode (PPN; *G. pallida*) colonised roots (PPN+AM fungi), and AM fungal-colonised roots that had PPN-infection on the opposing root compartment of the same host (PPN/AM fungi split roots). Letters indicate the significance in expression of that gene between the different treatments. N=4

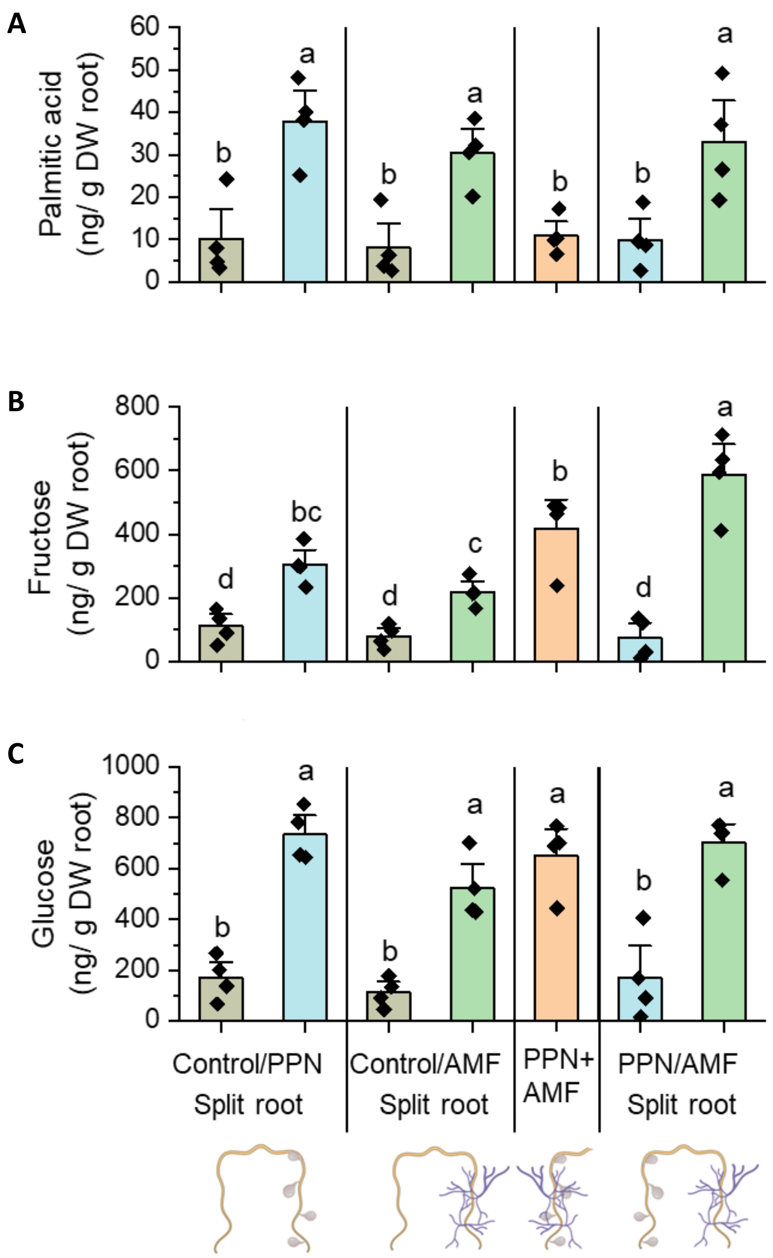

Fig S8: Relative concentrations of C16:0 palmitic acid, fructose and glucose within plant root tissues hyphae in presence and absence of plant parasitic nematodes (PPN) and arbuscular mycorrhizal fungal (AMF). Concentrations of (A) palmitic acid, (B) fructose, and (C) glucose in plant roots within each treatment (control = no symbiont, '/' = a single root system split between the two stated treatments, '+' = both symbionts together on the same roots). All data was collected at five weeks post inoculation. Bars represent standard error of the mean from four biological replicates. Different letters denote significance (Linear mixed-effects models were applied to account for the non-independency of split-roots belonging to the same plant, Tukey's Honest Significant Difference test, $p < 0.05$).

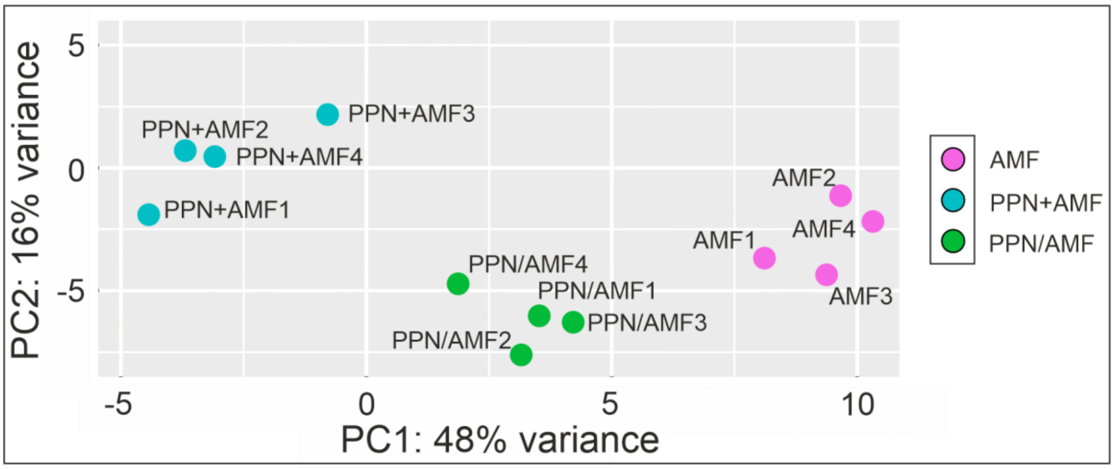

Fig S9. PCA plot of AM fungal gene expression from hosts co-inoculated with plant-parasitic nematodes. Data are gene expression profiles of AM fungi when no other symbionts are present on the host (AMF), when plant parasitic nematodes (PPN; *G. pallida*) were proximally infecting the roots (PPN+AMF), and when PPN were distally infecting the opposing root compartment (SplitAMF).

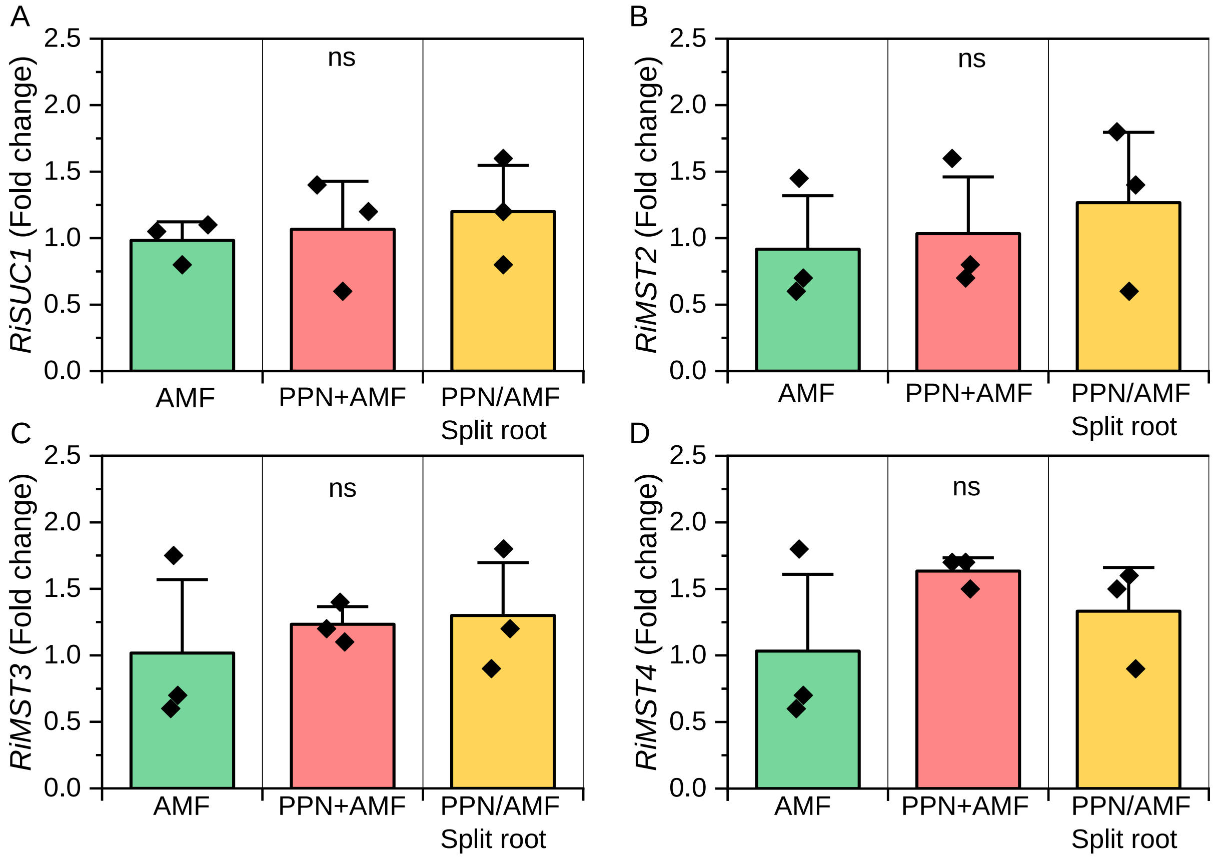

Fig S10: The expression of AM fungal genes related to monosaccharide assimilation from host plant roots across symbiont treatments. Expression (fold change relative to AM fungal treatment) of potential fungal sugar transporters (A-D) when plant parasitic nematodes (PPN; *G. pallida*) are on the same host in either the same (PPN+AMF) or separate (PPN/AMF) root compartments. Data were indicated by RNA-sequencing and confirmed via qPCR. Bars represent three biological replicates with standard error of the mean. ‘ns’ denotes no significance (one-way ANOVA, Tukey's Honest Significant Difference test, $p < 0.05$).

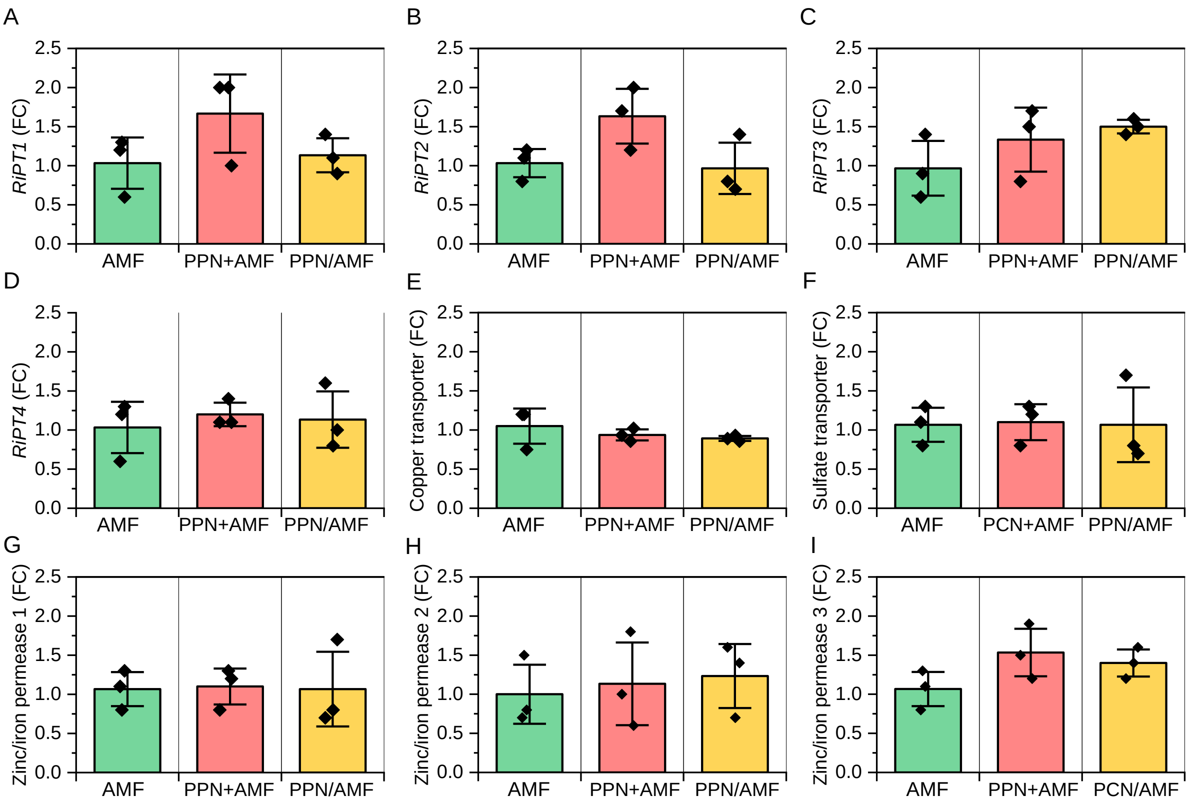

Fig S11: The expression of AM fungal genes related to nutrient transport in hosts with/without plant-parasitic nematode infection. Data indicates the expression (fold change relative to AM fungal treatment) of potential fungal nutrient transporters detected via root RNA-sequencing and confirmed via qPCR, when plant-parasitic nematodes (PPN; *G. pallida*) are on the same host in either the same (PPN+AMF) or separate (PPN/AMF) root compartments. Bars represent three biological replicates with standard error of the mean. Expression profiles of all genes were non-significant between treatments (one-way ANOVA, Tukey's Honest Significant Difference test, $p < 0.05$)

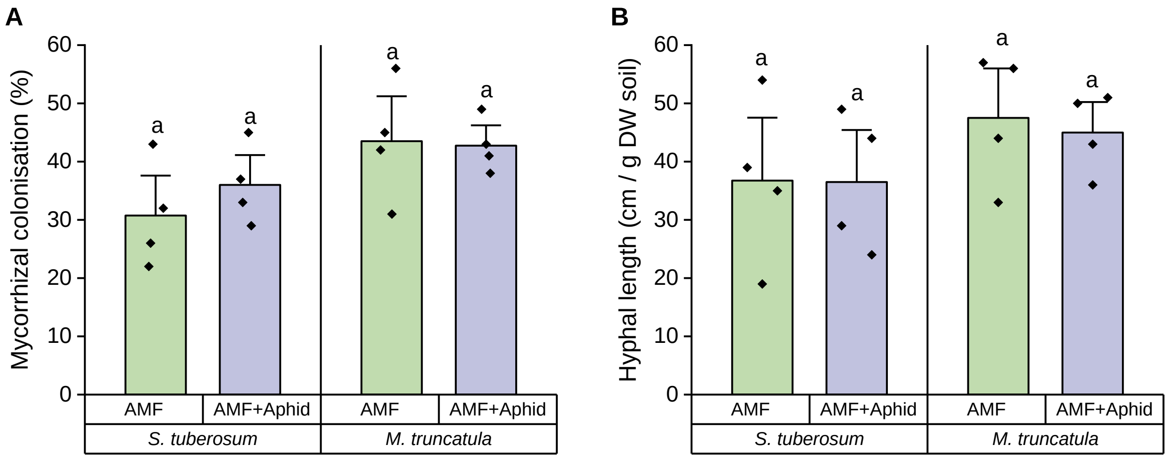

Fig S12. The impact of aphid herbivory on the colonisation of AM fungi on *S. tuberosum* and *M. truncatula* roots and soil. Data indicate (a) the percentage colonisation of *S. tuberosum* roots and (b) the hyphal lengths in the soil by AMF, either in the presence or absence of foliar-feeding aphids (*M. persicae*). Boxes represent four biological replicates and extend from the first to the third quartile with the middle bold line representing the median value, white circle representing the mean and whiskers extending to min/max. Different letters denote significance within plot panels (one-way ANOVA, Tukey's Honest Significant Difference test, $p < 0.05$).

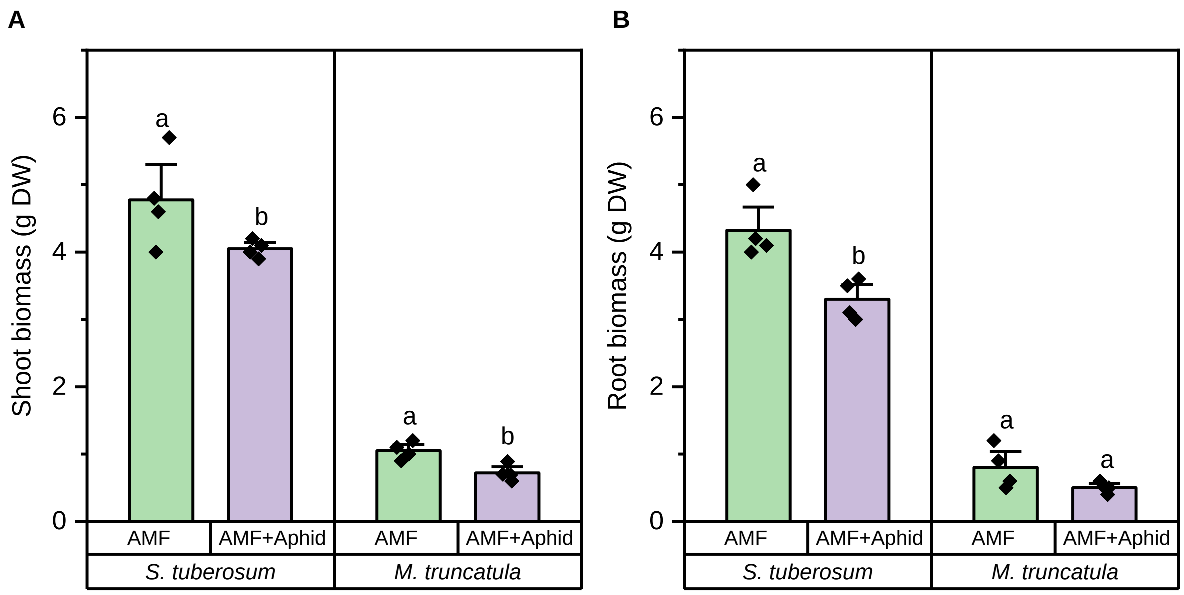

Fig S13. The impact of aphid herbivory on the shoot and root biomass of AM fungal-colonised *S. tuberosum* and *M. truncatula* hosts. Data indicate (a) shoot and (b) root dry weight of both *S. tuberosum* and *M. truncatula* plants colonised by AMF and either in the presence or absence of foliar-feeding aphids (*M. persicae*). Boxes represent four biological replicates and extend from the first to the third quartile with the middle bold line representing the median value, white circle representing the mean and whiskers extending to min/max. Different letters denote significance within plot panels (one-way ANOVA, Tukey's Honest Significant Difference test, $p < 0.05$).
