# Supplementary material for: Phytophagy impacts the quality and quantity of plant carbon resources acquired by mutualistic arbuscular mycorrhizal fungi
Source: Nat Commun. 2024 Jan 27;15:801. doi: 10.1038/s41467-024-45026-3 (PMC10821877; doi:10.1038/s41467-024-45026-3)
Supplement: Supplementary file 3 — Reporting Summary [file 41467_2024_45026_MOESM3_ESM.pdf]

Corresponding author(s): *Christopher Bell*

Last updated by author(s): *25/1023*

Reporting Summary

Nature Portfolio wishes to improve the reproducibility of the work that we publish. This form provides structure for consistency and transparency in reporting. For further information on Nature Portfolio policies, see our Editorial Policies and the Editorial Policy Checklist.

Statistics

For all statistical analyses, confirm that the following items are present in the figure legend, table legend, main text, or Methods section.

n/a	Confirmed	
☐	☒	The exact sample size (n) for each experimental group/condition, given as a discrete number and unit of measurement
☐	☒	A statement on whether measurements were taken from distinct samples or whether the same sample was measured repeatedly
☐	☒	The statistical test(s) used AND whether they are one- or two-sided<br>*Only common tests should be described solely by name; describe more complex techniques in the Methods section.*
☐	☒	A description of all covariates tested
☐	☒	A description of any assumptions or corrections, such as tests of normality and adjustment for multiple comparisons
☐	☒	A full description of the statistical parameters including central tendency (e.g. means) or other basic estimates (e.g. regression coefficient) AND variation (e.g. standard deviation) or associated estimates of uncertainty (e.g. confidence intervals)
☐	☒	For null hypothesis testing, the test statistic (e.g. F, t, r) with confidence intervals, effect sizes, degrees of freedom and P value noted<br>*Give P values as exact values whenever suitable.*
☒	☐	For Bayesian analysis, information on the choice of priors and Markov chain Monte Carlo settings
☒	☐	For hierarchical and complex designs, identification of the appropriate level for tests and full reporting of outcomes
☒	☐	Estimates of effect sizes (e.g. Cohen's d, Pearson's r), indicating how they were calculated

Our web collection on statistics for biologists contains articles on many of the points above.

Software and code

Policy information about availability of computer code

Data collection	*No software was used to collect data in this study.*
Data analysis	As mentioned in the Methods, the following software were used to analyse data in this study: Cyclone Plus, TraceFinder, Trimmomatic, HISAT2.2.1, HTSeq 2.05, DESeq2 1.42, EdgeR 4.0.5 , Omicsbox BLAST2GO 3.1, Biorad CFX Manager 3.1, OriginPro 2020 9.7.0.188, RStudio 2023.06.1 Build 524.<br><br>"lmerTest 3.1" and "emmeans 1.9" packages were used within R v4.03.

For manuscripts utilizing custom algorithms or software that are central to the research but not yet described in published literature, software must be made available to editors and reviewers. We strongly encourage code deposition in a community repository (e.g. GitHub). See the Nature Portfolio guidelines for submitting code & software for further information.

Data

Policy information about availability of data

All manuscripts must include a data availability statement. This statement should provide the following information, where applicable:

- Accession codes, unique identifiers, or web links for publicly available datasets
- A description of any restrictions on data availability
- For clinical datasets or third party data, please ensure that the statement adheres to our policy

The data generated during transcriptomic analyses of symbiont-interacting root tissues of Solanum tuberosum are available in the SRA database under PRJNA1005093. The datasets generated during other experiments from the current study are available from the corresponding author on reasonable request.

Research involving human participants, their data, or biological material

Policy information about studies with human participants or human data. See also policy information about sex, gender (identity/presentation), and sexual orientation and race, ethnicity and racism.

Reporting on sex and gender	*No human data were collected.*
Reporting on race, ethnicity, or other socially relevant groupings	*No human data were collected.*
Population characteristics	*No human data were collected.*
Recruitment	*No human data were collected.*
Ethics oversight	*No human data were collected.*

Note that full information on the approval of the study protocol must also be provided in the manuscript.

Field-specific reporting

Please select the one below that is the best fit for your research. If you are not sure, read the appropriate sections before making your selection.

☒ Life sciences ☐ Behavioural & social sciences ☐ Ecological, evolutionary & environmental sciences

For a reference copy of the document with all sections, see nature.com/documents/nr-reporting-summary-flat.pdf

Life sciences study design

All studies must disclose on these points even when the disclosure is negative.

Sample size	*No sample size calculations were performed. Sample sizes were determined by technical tractability. 1) Radioisotope usage is stringently controlled and a reduction in usage is emphasised under local regulations, therefore minimum replicates based on prior in-house experiments informed replicate size. 2) The extremely challenging collection of hyphal material placed an upper threshold on replicate size. 3) Technical replicates were used where possible to ensure each biological replicate was representative*
Data exclusions	*No data were excluded.*
Replication	*All collected data that expand upon prior publications (e.g. https://doi.org/10.1111/nph.17958) corroborate prior datasets, therefore exhibit successful replication.*
Randomization	*The fungal spores and nematode cysts were collected from separate pooled stocks that was mixed prior to removal of inocula to ensure random selection of individuals. Apterous aphid individuals were randomly collected from culture plants for inoculation onto experimental plants.*<br>*therefore were randomly selected for each treatment replicate. All glasshouse pots were randomised.*
Blinding	*The investigators were not blind to treatment allocation during data collection or analyses as inoculated organisms were collected upon harvest, thereby indicating treatments.*

Behavioural & social sciences study design

All studies must disclose on these points even when the disclosure is negative.

Study description	*N/A*

Research sample	N/A
Sampling strategy	N/A
Data collection	N/A
Timing	N/A
Data exclusions	N/A
Non-participation	N/A
Randomization	N/A

Ecological, evolutionary & environmental sciences study design

All studies must disclose on these points even when the disclosure is negative.

Study description	N/A
Research sample	N/A
Sampling strategy	N/A
Data collection	N/A
Timing and spatial scale	N/A
Data exclusions	N/A
Reproducibility	N/A
Randomization	N/A
Blinding	N/A

Did the study involve field work? ☐ Yes ☐ No

Field work, collection and transport

Field conditions	N/A
Location	N/A
Access & import/export	N/A
Disturbance	N/A

Reporting for specific materials, systems and methods

We require information from authors about some types of materials, experimental systems and methods used in many studies. Here, indicate whether each material, system or method listed is relevant to your study. If you are not sure if a list item applies to your research, read the appropriate section before selecting a response.

Materials & experimental systems

n/a	Involved in the study
☒	☐ Antibodies
☒	☐ Eukaryotic cell lines
☒	☐ Palaeontology and archaeology
☐	☒ Animals and other organisms
☒	☐ Clinical data
☒	☐ Dual use research of concern
☐	☒ Plants

Methods

n/a	Involved in the study
☒	☐ ChIP-seq
☒	☐ Flow cytometry
☒	☐ MRI-based neuroimaging

Antibodies

Antibodies used: *N/A*

Validation: *N/A*

Eukaryotic cell lines

Policy information about cell lines and Sex and Gender in Research

Cell line source(s): *N/A*

Authentication: *N/A*

Mycoplasma contamination: *N/A*

Commonly misidentified lines (See ICLAC register): *N/A*

Palaeontology and Archaeology

Specimen provenance: *N/A*

Specimen deposition: *N/A*

Dating methods: *N/A*

☐ Tick this box to confirm that the raw and calibrated dates are available in the paper or in Supplementary Information.

Ethics oversight: *N/A*

Note that full information on the approval of the study protocol must also be provided in the manuscript.

Animals and other research organisms

Policy information about studies involving animals; ARRIVE guidelines recommended for reporting animal research, and Sex and Gender in Research

Laboratory animals: *Globodera pallida (Lindley) cysts. Myzus persicae (Clone O) apterous individuals (approximately 7 days old).*

Wild animals: *This study did not involve wild animals.*

Reporting on sex: *Sex was not relevant in this study design.*

Field-collected samples: *No field samples were used in this study. Nematodes were reared on Solanum tuberosum (Desiree) at 19C 16hr days for 12 weeks. Soils were then colected and stored at 4C prior to the extraction of nematode cysts and use in this study. Aphids were reared on S. tuberosum plants at 19C 16hr days until hand-picking individuals for this study.*

Ethics oversight: *No ethical approval is required for the animals selected in this study.*

Note that full information on the approval of the study protocol must also be provided in the manuscript.

Clinical data

Policy information about clinical studies

All manuscripts should comply with the ICMJE guidelines for publication of clinical research and a completed CONSORT checklist must be included with all submissions.

Clinical trial registration	*N/A*
Study protocol	*N/A*
Data collection	*N/A*
Outcomes	*N/A*

Dual use research of concern

Policy information about dual use research of concern

Hazards

Could the accidental, deliberate or reckless misuse of agents or technologies generated in the work, or the application of information presented in the manuscript, pose a threat to:

No	Yes	
☒	☐	Public health
☒	☐	National security
☒	☐	Crops and/or livestock
☒	☐	Ecosystems
☒	☐	Any other significant area

Experiments of concern

Does the work involve any of these experiments of concern:

No	Yes	
☒	☐	Demonstrate how to render a vaccine ineffective
☒	☐	Confer resistance to therapeutically useful antibiotics or antiviral agents
☒	☐	Enhance the virulence of a pathogen or render a nonpathogen virulent
☒	☐	Increase transmissibility of a pathogen
☒	☐	Alter the host range of a pathogen
☒	☐	Enable evasion of diagnostic/detection modalities
☒	☐	Enable the weaponization of a biological agent or toxin
☒	☐	Any other potentially harmful combination of experiments and agents

Plants

Seed stocks	Solanum tuberosum seed potatoes variety Desiree were obtained from Denholm Seed Potatoes, Scotland, UK. Medicago truncatula A17 seeds are from a lab collection.
Novel plant genotypes	No novel plant genotypes were created in this study.
Authentication	*No authentication of seed stock was required.*

ChIP-seq

Data deposition

☐ Confirm that both raw and final processed data have been deposited in a public database such as GEO.

☐ Confirm that you have deposited or provided access to graph files (e.g. BED files) for the called peaks.

Data access links
May remain private before publication.

N/A

Files in database submission

N/A

Genome browser session
(e.g. UCSC)

N/A

Methodology

Replicates: N/A

Sequencing depth: N/A

Antibodies: N/A

Peak calling parameters: N/A

Data quality: N/A

Software: N/A

Flow Cytometry

Plots

Confirm that:

- [ ] The axis labels state the marker and fluorochrome used (e.g. CD4-FITC).
- [ ] The axis scales are clearly visible. Include numbers along axes only for bottom left plot of group (a 'group' is an analysis of identical markers).
- [ ] All plots are contour plots with outliers or pseudocolor plots.
- [ ] A numerical value for number of cells or percentage (with statistics) is provided.

Methodology

Sample preparation: N/A

Instrument: N/A

Software: N/A

Cell population abundance: N/A

Gating strategy: N/A

- [ ] Tick this box to confirm that a figure exemplifying the gating strategy is provided in the Supplementary Information.

Magnetic resonance imaging

Experimental design

Design type: N/A

Design specifications: N/A

Behavioral performance measures: N/A

Acquisition

Imaging type(s): N/A

Field strength: N/A

Sequence & imaging parameters: N/A

Area of acquisition: N/A

Diffusion MRI ☐ Used ☐ Not used

Preprocessing

Preprocessing software	*N/A*
Normalization	*N/A*
Normalization template	*N/A*
Noise and artifact removal	*N/A*
Volume censoring	*N/A*

Statistical modeling & inference

Model type and settings	*N/A*
Effect(s) tested	*N/A*

Specify type of analysis: ☐ Whole brain ☐ ROI-based ☐ Both

Statistic type for inference (See Eklund et al. 2016)	*N/A*
Correction	*N/A*

Models & analysis

n/a	Involved in the study
☐	☐ Functional and/or effective connectivity
☐	☐ Graph analysis
☐	☐ Multivariate modeling or predictive analysis

Functional and/or effective connectivity	*N/A*
Graph analysis	*N/A*
Multivariate modeling and predictive analysis	*N/A*
